# Efficient single nucleotide polymorphism discovery in laboratory rat strains using wild rat-derived SNP candidates

**DOI:** 10.1186/1471-2164-6-170

**Published:** 2005-11-29

**Authors:** Bart MG Smits, Victor Guryev, Dimphy Zeegers, Dirk Wedekind, Hans J Hedrich, Edwin Cuppen

**Affiliations:** 1 Hubrecht Laboratory, Functional Genomics Group, Uppsalalaan 8, 3584 CT Utrecht, The Netherlands; 2 Institute for Laboratory Animal Science, Hannover Medical School, Carl-Neuberg-Str.1, 30625 Hannover, Germany

## Abstract

**Background:**

The laboratory rat (*Rattus norvegicus*) is an important model for studying many aspects of human health and disease. Detailed knowledge on genetic variation between strains is important from a biomedical, particularly pharmacogenetic point of view and useful for marker selection for genetic cloning and association studies.

**Results:**

We show that Single Nucleotide Polymorphisms (SNPs) in commonly used rat strains are surprisingly well represented in wild rat isolates. Shotgun sequencing of 814 Kbp in one wild rat resulted in the identification of 485 SNPs as compared with the Brown Norway genome sequence. Genotyping 36 commonly used inbred rat strains showed that 84% of these alleles are also polymorphic in a representative set of laboratory rat strains.

**Conclusion:**

We postulate that shotgun sequencing in a wild rat sample and subsequent genotyping in multiple laboratory or domesticated strains rather than direct shotgun sequencing of multiple strains, could be the most efficient SNP discovery approach. For the rat, laboratory strains still harbor a large portion of the haplotypes present in wild isolates, suggesting a relatively recent common origin and supporting the idea that rat inbred strains, in contrast to mouse inbred strains, originate from a single species, *R. norvegicus*.

## Background

Genetic variation exists between individuals (or strains) of all organisms and it makes up the genetic basis for phenotypic differences between individuals. In addition, genetic variation functions as a valuable resource for mapping phenotypic traits in model organisms. Single Nucleotide Polymorphisms (SNPs) are the most abundant form of genetic variation and therefore dominate high-resolution genetic mapping strategies. Moreover, numerous well-performing high-throughout SNP detection technologies have been developed, like oligonucleotide array-based technology, mass-spectrometry-based technology (MALDI-TOF), and sequence-based technology (pyrosequencing, DHPLC) [1], which makes automated SNP detection favored above the more labor-intensive detection of microsatellite markers [2].

Since the availability of its genome, the laboratory rat is gaining influence as a genetic model organism [3]. In addition, over 200 well-characterized inbred strains that are models for a wide variety of human diseases are available [4,5]. However, the availability of genetic tools, like a dense genome-wide SNP marker set, is still subordinate compared to other commonly used model organisms. This is illustrated by the number of entries in dbSNP, the central SNP repository of NCBI [6]: the amount of human (>10,000,000), chicken (>3,000,000), and mouse (>500,000) entries surpass the amount of rat entries (>43,000) spectacularly. In search for rat SNPs, experimental [7,8] and computational [9] approaches have been employed, but these efforts primarily resulted in SNPs associated with coding regions. For genetic mapping purposes, a much denser marker set, preferentially equally distributed over the genome, is required.

Laboratory rat strains are thought to be established from a limited number of founder animals originating from a domesticated wild population [10,11]. The value of inbred strains emanates from the close genetic uniformity that facilitates phenotyping and genotyping. In principle, inbred strains are selectively bred for certain traits from a genetically diverse pool, comprising diverse genetic information about the trait. However, since many of the current rat strains were derived from common ancestral stocks and simply inbred to increase genetic uniformity, inbred strains clearly share alleles [12]. Although such simplified models are essential for biomedical research, modulating effects on the clinical manifestation of a trait resulting from genetic heterogeneity in a population can only be studied to a limited extent in F1 hybrids. The use of a carefully chosen selection of inbred strains may address this issue, but the choice depends on knowledge on the relationship between the strains and hence the degree of genetic variation. Alternatively, wild-derived strains may be good alternatives to introduce sufficient genetic variation in laboratory experiments [13,14].

Based on a preliminary observation that alleles from laboratory rat strains are frequently detected in wild-derived samples, we developed a wild rat-based SNP discovery approach. The method consists of shotgun sequencing of a wild rat-derived genomic library followed by comparison with the published rat genome (strain Brown Norway). Genotyping commonly used rat strains for newly identified SNPs revealed that 84% of SNP-alleles (and 87% of all genetic variation) occurring between BN and a single wild individual is also represented in one or more laboratory strains. A user-friendly webtool allows exploration of the genetic variation between any arbitrary combinations of two strains that were used in this study, making all information directly available for experimental use.

## Results

### Wild rat-based SNP discovery

It is generally believed that commonly used rat strains originate from a wild-derived founder population of limited size [10]. To examine whether polymorphisms found in laboratory strains are still represented in individuals of the wild population, we typed two wild-derived samples for confirmed SNPs of the CASCAD database [9]. Interestingly, about 53% of alleles (n = 147), which were confirmed to exist in laboratory strains, were also represented in wild 1, wild 2 or both **(**not shown). Hence, a preselection of highly likely candidate SNPs could potentially be made by genotyping wild individuals and comparing the sequences to the rat genome sequence (Brown Norway).

Accordingly, we performed random shotgun sequencing on a genomic library of a wild rat (wild 1). We generated shotgun traces (814 Kbp) by bidirectional sequencing of about 1,600 colonies (Table 1). 85.5% of the reads (2545/2975; Table 1) could be mapped to a unique location in the Brown Norway rat genome using BLAT [15], resulting in the automated identification of nearly 5,000 ambiguous nucleotide positions (potential polymorphisms). Manual inspection of the sequencing reads reduced this set of potential polymorphisms to a set of 746 real SNPs and 122 indels. The average SNP rate between BN (BN/SsNMcw; genome sequencing project) and this single wild rat is estimated to be about 1 per 900 bp and, hence, discovery of a novel SNP can be expected every second shotgun read. A subset of the discovered SNPs was verified and genotyped in 36 commonly used strains (including BN). To this end, we designed primers for 451 SNP-containing amplicons (about 300 bp) of which 416 (92.2%) were successfully read by unidirectional sequencing of the PCR products, resulting in roughly 119 Kbp high quality sequence per strain or individual (Table 1).

**Table 1:** Statistics on shotgun sequencing of the wild rat-derived genomic library

picked colonies	1632
readable sequence reads/sequenced bases	2975/814,440
uniquely mapped (BLAT) reads/bases	2545/768,683
ambiguous positions	4902
strong candidates after manual inspection	868 (746 SNPs + 122 indels)
successfully read/amplicons designed*	416/451 (~1.65 candidate SNP/amplicon) (92.2%)
amplified bases per strain or wild individual	118,971

* Amplicons are designed for the 746 SNP candidates.

### Wild rat-derived SNP characteristics

**Figure 1:**
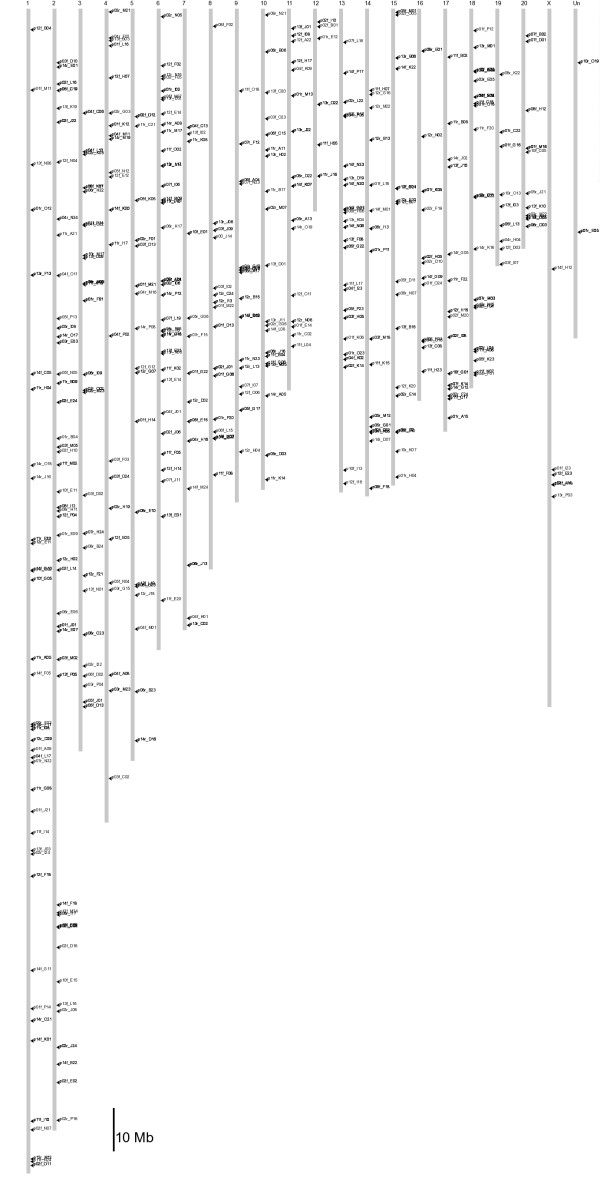
Distribution of amplicons (451 loci) designed for verification and subsequent genotyping of candidate shotgun-based SNPs in 36 commonly used inbred strains.

The verification of 746 candidate SNPs by amplicon-based resequencing in 36 inbred rat strains and three wild-derived samples (wild 1, 2, and 3) revealed 960 polymorphisms, consisting of 90 indels, seven 2-bp substitutions, one 3-bp substitution, one 5-bp substitution, and 861 SNPs, of which only one was tri-allelic. The amplicons are randomly distributed over the genome (Fig. 1). We observed heterozygous positions in the outbred strains, but unexpectedly some were also found in the inbred strains (for detailed information: [see Additional file 1] or [6]). For our analysis, we considered these loci to be polymorphic as compared to the BN genome sequence.

From the 746 shotgun-based candidate SNPs, 685 were located in the 416 PCR amplicons that worked, and 485 (71%) were reconfirmed by resequencing (shotgun-based; Table 2). Strikingly, for 408 (84%) of the confirmed SNPs, the wild rat allele is also present in one or more commonly used strains, with only 36 (7.4%) being specific to BN (Table 2). Of the remaining 77 (16%) SNPs, wild rat alleles are not present in any of the 36 selected strains and could be considered wild rat-specific. These results illustrate that shotgun sequencing one wild individual efficiently identifies shared polymorphisms among commonly used rat strains.

**Table 2:** SNP discovery results

	shotgun- based	genotyping-based (only wild 1*)	genotyping-based (wild 1, 2, 3*)	total (only wild 1*)	total (wild 1, 2, 3*)
BN specific	36 (7.4 %)	9 (2.5 %)	7 (1.9 %)	45 (5.3 %)	43 (5.0 %)
wild specific	77 (15.9 %)	12 (3.4 %)	30 (8.0 %)	89 (10.6 %)	107 (12.4 %)
in 35 strains, not in wild	0	204 (57.0 %)	156 (41.5 %)	204 (24.2 %)	156 (18.1 %)
in 35 strains, shared with wild	372 (76.7 %)	133 (37.1 %)	183 (48.7 %)	505 (59.9 %)	555 (64.5 %)

total	485 (100 %)	358 (100 %)	376 (100 %)	843 (100 %)	861 (100 %)

* By genotyping two other wild individuals (wild 2 and 3), additional polymorphisms were identified, which could not have been found by shotgun sequencing only wild 1.

While genotyping by resequencing, 358 novel SNPs were discovered that were not identified in the shotgun sequencing experiment (genotyping-based; Table 2). About 39% (139) of this set can be accounted for by differences in the sequence coverage between the shotgun reads and the resequencing genotyping reads (Table 2), whereas the remaining part of this set is strongly biased towards SNPs that are not polymorphic between BN and wild rat 1 and thus could not have been discovered in the shotgun experiment. Interestingly, about 37% of the newly discovered SNPs are polymorphic between the shotgun sequenced wild rat and any of the inbred strains (Table 2). When considering all SNPs that are polymorphic in the set of 36 commonly used laboratory strains, of the majority (66%) the wild rat allele is found back in one of the strains (total; Table 2) and this percentage increases only slightly (70%) when two other wild individuals (wild 2 and 3) are included in the analysis. This indicates that wild rat-based SNP discovery is already highly efficient using a single wild sample.

Based on the genotyping results, the SNP rate between BN and the shotgun sequenced wild rat (wild 1) is 1 SNP per 190 bp (626 SNPs/119 Kbp). The SNP rate within the 36 rat strains, including BN, is 1 in 158 (Table 2; 45+204+505 SNPs/119 Kbp) and the SNP rate in the entire experiment, including the wild rat (wild 1), BN, and the other strains is 1 in 141 bp (Table 2; 843 SNPs/119 Kbp). To compare wild rat inter-individual variation with the inter-strain variation for commonly used inbred strains, we calculated the number of SNPs that are polymorphic when comparing arbitrary combinations of 3 strains. Genotyping of 861 SNP positions in the three wild rats resulted in 438 polymorphic positions, whereas the most polymorphic combination of inbred strains in this experiment (BN, BH, and SHR) yielded 427 SNPs. This indicates that three random, but potentially related, Dutch wild rats are about equally polymorphic as three carefully selected inbred strains. Inclusion of wild isolates from other locations worldwide may increase the efficiency of the SNP discovery approach.

### Intraspecific phylogenetic network

**Figure 2:**
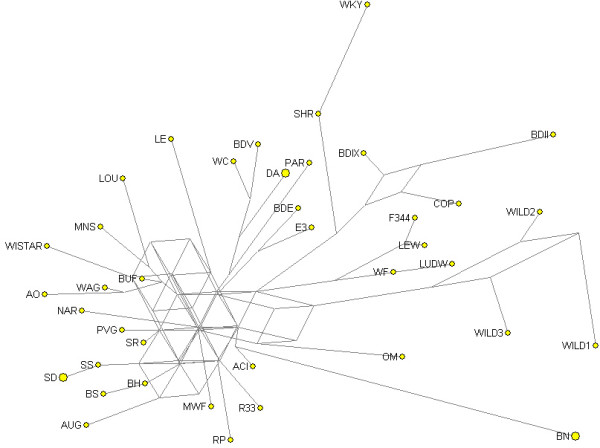
Strain relationships in a network structure. End nodes (yellow dots) represent strains. Some end nodes are double-size, meaning that they are supported by two samples. Interconnecting nodes where lines come together, represent a possible precursor.

Relationships among different rat strains have been determined previously by phylogenetic tree reconstruction based on microsatellite markers [16,17]. However, intraspecific relationships for laboratory strains are often very challenging to determine, due to small genetic distances and complex gene flow. The resulting multitude of plausible trees is best expressed by a network, which displays alternative potential evolutionary paths in the form of cycles [18]. We used Network software (v4.111 Reduced-Joining, [19]) to construct a spatial network, based on 861 SNP markers in 36 rat strains and three wild rat individuals (Fig. 2). The three wild individuals are grouped together, possibly due to the geographic and possibly genetic relation between the samples, but in accordance with the last paragraph of the previous section, they appear relatively unrelated as compared to the set of inbred strains.

The majority of the SNPs (485 of 861) was selected for being polymorphic between wild 1 and BN. As a result, different BN substrains (BN/Ztm, BN/Crl), depicted as a double-sized end node because of high similarity, and different wild rat individuals (wild 1, wild 2, and wild 3) are grouped together as the outliers. Several strains that are known to be closely related (source RGD-strains: [20]) are also grouped together, like DA and COP or SS and SR. Interestingly, WKY is also an outlier, indicating that besides BN, this strain can be utilized as an alternative mapping strain. WKY is already commonly used as a normotensive control strain in genetic mapping of blood pressure quantitative trait loci [21]. WKY is known to be closely related to SHR and these strains are indeed grouped together (Fig. 2). Additionally, BDII and BDIX are related and BDE is an RI strain from E3. These strain combinations are also grouped together. Wistar is contributing to a large subset of these strains, like WKY, WC, BDII, MWF, LEW, and WF, which contributes to the complexity of the network structure.

### Data availability

The use of genetic markers for mapping traits in rat strains has been exploited for long time already. Current marker sets in rats are mostly limited to microsatellites [22,23], which are not abundantly available and are commonly detected in a more laborious way than SNPs. In this study, we have determined a total of about 35,000 genotypes (about 960 loci in 36 inbred strains), out of which the vast majority are SNPs. This data is accessible via a versatile webtool [24]. Pairs of strains of interest can be selected and explored on presence of verified genetic variation. Besides a graphical representation of the location of the SNPs on a genome map, primer sequences that were successfully used in our experiments are also provided. In a pairwise comparison matrix (Table 3), we plotted the absolute number of polymorphic positions for each of the (sub-)strains or individuals used. Interestingly, for some strains different alleles are observed in substrains (e.g. BN/Crl differs from BN/Ztm at 4 positions), in line with previous observations [8].

**Table 3:** Absolute number of polymorphic positions between strains in a pairwise comparison.

	ACI	AO	AUG	BDE	BDII	BDIX	BDV	BH	BN	BN2	BS	BUF	COP	DA	DA2	E3	F344	LEW	LE	LOU	LUDW	MWF	MNS	NAR	OM	PAR	PVG	R33	RP	SD	SD2	SHR	SR	SS	WAG	WC	WF	WIST	WKY	wild3	wild2	wild1
ACI	x																																									
AO	131	x																																								
AUG	111	167	x																																							
BDE	145	178	158	x																																						
BDII	130	166	143	148	x																																					
BDIX	129	196	191	159	115	x																																				
BDV	109	131	145	137	78	142	x																																			
BH	145	189	192	212	156	177	167	x																																		
BN	225	263	279	266	206	258	244	274	x																																	
BN2	227	270	285	268	222	271	251	274	4	x																																
BS	159	167	163	178	166	185	143	180	246	251	x																															
BUF	148	158	181	171	168	190	151	166	250	263	158	x																														
COP	84	194	187	166	155	147	155	191	262	275	203	192	x																													
DA	62	107	116	134	115	130	117	130	210	216	150	126	116	x																												
DA2	76	141	144	157	149	167	127	176	261	269	193	167	151	2	x																											
E3	136	189	170	86	154	180	144	204	244	253	172	182	171	148	180	x																										
F344	132	165	178	178	152	150	138	144	170	176	141	156	166	121	165	168	x																									
LEW	156	178	213	197	160	163	141	169	213	222	166	160	191	133	181	186	16	x																								
LE	131	144	136	155	148	152	122	142	221	224	157	127	147	110	140	167	142	149	x																							
LOU	145	146	165	191	153	203	120	178	242	250	138	171	212	111	142	192	126	150	149	x																						
LUDW	153	175	186	198	161	169	153	195	252	263	183	177	189	123	163	215	115	125	133	161	x																					
MWF	148	147	172	166	136	166	111	167	209	222	135	148	185	133	164	158	115	134	148	136	163	x																				
MNS	151	167	178	173	158	186	122	169	239	250	155	194	210	128	176	166	123	132	156	141	159	137	x																			
NAR	147	169	184	212	168	177	145	177	233	249	146	155	197	134	193	188	137	166	122	170	153	164	151	x																		
OM	127	161	153	170	120	158	143	150	216	222	154	138	176	125	147	183	125	156	127	156	139	143	144	147	x																	
PAR	140	182	168	166	149	158	133	175	225	227	155	138	167	136	159	169	128	136	127	151	159	133	160	150	162	x																
PVG	95	164	152	153	153	181	129	184	252	263	170	151	164	110	150	148	142	175	145	143	196	146	161	172	160	147	x															
R33	155	198	183	213	169	198	186	177	261	257	173	210	204	142	184	223	183	209	164	185	196	191	187	182	159	173	189	x														
RP	146	159	171	171	132	164	109	186	216	230	113	161	176	138	175	153	119	141	134	141	166	108	157	136	147	132	139	166	x													
SD	121	154	156	177	153	160	129	118	233	247	149	134	174	100	148	184	131	138	135	125	145	144	141	149	103	133	130	147	138	x												
SD2	95	116	134	150	117	130	122	85	219	220	117	96	145	104	115	168	109	115	92	99	110	118	121	107	90	121	109	126	109	16	x											
SHR	159	212	166	186	179	175	168	204	264	275	188	178	180	135	189	205	156	176	173	202	188	180	200	184	182	177	192	207	187	185	139	x										
SR	129	171	172	170	160	161	147	138	235	244	163	131	170	120	172	174	146	169	156	157	175	150	160	164	136	117	134	166	160	60	64	184	x									
SS	114	145	167	183	142	150	139	111	249	253	149	152	175	117	161	186	136	161	121	134	156	146	153	128	130	143	145	145	144	69	46	191	83	x								
WAG	120	105	156	147	143	158	115	160	197	200	96	125	164	94	136	151	110	132	127	96	145	120	121	129	140	126	128	154	108	118	84	181	119	110	x							
WC	140	164	158	155	129	180	92	188	214	232	151	177	177	125	158	157	131	160	156	145	171	87	157	179	152	158	138	213	126	153	122	195	170	157	126	x						
WF	155	183	195	211	162	164	152	183	266	274	176	173	193	129	175	224	120	123	133	156	50	180	158	148	141	155	196	189	157	141	97	201	179	135	149	186	x					
WIST	101	116	116	113	98	101	112	93	160	162	107	91	119	83	114	133	82	86	82	110	95	89	94	85	105	101	96	115	100	83	51	112	71	67	76	114	91	x				
WKY	169	208	198	213	164	188	168	208	264	276	208	181	205	128	170	228	180	210	165	229	206	200	220	195	189	197	208	205	194	196	148	115	196	189	183	216	203	101	x			
wild3	137	181	153	177	162	164	134	149	175	194	156	173	153	111	172	169	162	182	156	184	173	171	160	149	134	140	161	154	165	161	120	152	160	152	140	158	178	108	163	x		
wild2	197	233	207	223	182	213	194	187	256	268	203	221	211	181	227	218	204	243	180	234	220	213	210	203	177	198	219	202	214	210	163	190	208	205	208	213	210	141	210	52	x	
wild1	334	414	368	406	329	405	339	372	520	551	369	400	395	315	392	397	386	446	338	415	410	413	391	403	331	352	377	371	387	373	321	334	375	388	372	404	410	280	357	134	157	x

The matrix is built from genotyping data of 960 polymorphisms in 36 strains and three wild individuals. Two inbred strains are represented by two substrains (BN and DA) and outbred SD is represented by two individuals from different stocks. Sets of polymorphisms, including a graphical representation, can be retrieved from [24].

### Simulation experiment wild rat-based SNP discovery

**Figure 3:**
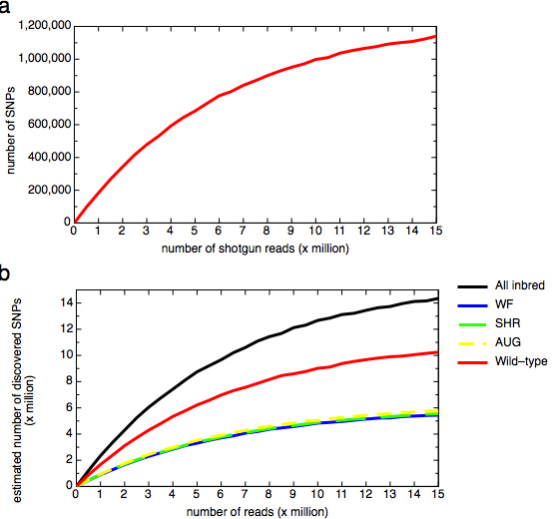
**a) **Simulation of wild rat-based SNP discovery experiment. Simulation is based on the discovery of 485 SNPs between wild 1 and BN in 814 Kbp of shotgun sequence. For 408 of those, the wild rat alleles is found back in one or more inbred strains. The relation between generation of randomly distributed 400 bp shotgun reads and estimated number of newly discovered SNPs is plotted. **b) **Simulation of SNP discovery experiment, using carefully selected (most polymorphic compared to BN) rat strains (SHR, AUG, and WF) or all rat strains, in comparison with wild rat-based SNP discovery. Simulation is based on 539, 304, 292, 287, and 754 SNPs for wild 1, AUG, SHR, WF, and all strains respectively, in 119 Kbp of genotyped sequence.

To get insight in the benefits of using wild rats in SNP discovery studies, we simulated larger scale experiments based on the results obtained in the experiments described above. Shotgun sequencing of 814 Kbp resulted in the identification of 485 SNPs. For 408 of those, the wild rat allele was also represented in laboratory rat strains and hence of interest for research purposes. The maximum amount of SNPs that can be discovered by fully sequencing this single rat is calculated by multiplying the SNP frequency (408/814,440) with the rat genome size (2,48 Gbp), which is 1,252,911 SNPs. Since none of our shotgun reads were overlapping, we can calculate the relation between shotgun sequencing reads of the wild rat and the amount of SNPs that will be found by scaling up this methodology, assuming random distribution of 400 bp shotgun reads over the genome (Fig. 3a). One million shotgun reads of a single wild rat would already result in the discovery of 200,000 novel SNPs that are polymorphic in commonly used rat strains. This simulation indicates that a relatively small sequencing effort could potentially result in a vast expansion of the amount of genetic variation for the rat.

Because shotgun sequencing was only done in the wild rat 1, we cannot make a direct comparison between wild rat-based SNP discovery and SNP discovery based on rat strains separately. However, a similar simulation experiment can be performed by treating the genotyping resequencing as shotgun reads. For wild 1, this would result in the identification of 577 SNPs as compared to the BN genome sequence. For 539 of those, the wild rat allele is found back in one of the inbred strains. For the combination of three strains most polymorphic as compared to BN in this experiment, the latter number would be 304, 292, and 287 for AUG, SHR, and WF, respectively. Simulations based on these numbers show that it requires nearly two times as much shotgun sequencing in different inbred strains separately to discover the same amount of SNPs that can be found using the wild rat shotgun sequencing approach. It should be mentioned that parallel shotgun sequencing of all 36 inbred strains until saturation has the potential to yield 1.6 times as many SNPs as compared to the wild-derived approach (Fig. 3b). An advantage of using inbred strains for SNP discovery is that the genotype of the strain is immediately known. Nevertheless, reconfirmation of the SNP or genotyping of other strains of interest may be necessary anyway, minimizing the relevance of this advantage.

## Discussion

An increase in the amount of documented genetic variation for the rat will be essential to allow for high-resolution genetic mapping of the many inherited traits that have now been described for a wide variety of rat inbred strains. In addition, insight into genetic variation between rat strains provides valuable information on genetic relationships between strains, which can be instrumental to dissect the genetic basis of phenotypic differences. The wild rat-based shotgun sequencing method described here provides an efficient approach to generate such a dense map of genetic variation. To be able to benefit from haplotype-based mapping approaches [25-28] a high marker density is needed to first reliably define haplotype blocks in strains of interest [29]. For the mouse, it has recently been announced that 15 inbred strains will be fully resequenced to achieve this goal [30]. With extreme dense genotype maps, it may even become possible to clone traits by haplotype-based *in silico *mapping [25], but to achieve this, it is estimated that complete sequences of over 50 strains are needed [29]. Although densities needed for these approaches are not reached, we do show here that wild rat-based SNP discovery is potentially much more effective than shotgun sequencing different inbred strains. We propose that the most effective SNP discovery strategy for the rat would be one based on shotgun sequencing of a single wild-derived sample and subsequent low-cost high-throughput genotyping of the resulting candidates in the laboratory strains of interest. Many other model organisms are currently undergoing full coverage sequencing and SNP discovery in these organisms will become increasingly important, especially for those organisms that are selectively bred for specific traits, such as cow and pig. Pilot experiments using for example wild-derived swine samples could be performed to test whether it is eligible to efficiently transfer the wild isolate-based SNP discovery strategy to other organisms.

Our results do provide insight in the genetic descent of the laboratory rat. It is generally accepted that current rat strains underwent two major genetic bottlenecks. First, they originate from a small founder population of domesticated wild rats and second, they were selectively inbred to obtain homogeneity [11]. The three Dutch wild rats used in this study are potentially relatively closely related as compared to wild rats from different parts of the world, but the genetic variation between them is mostly larger than or sporadically equal to any combination of three inbred strains, indeed suggesting the existence a common genetic bottleneck for laboratory strains. In addition, the laboratory rat does not show an extensive polymorphism rate in the MHC (major histocompatibilty complex) as compared to other species [31], like human, cattle etc. Cramer et al. has analyzed the MHC of wild rats and compared the data with those from inbred strains [32]. In line with our observation, there were not many new haplotypes.

We observed that wild rat genetic variation is to a large extent represented in the inbred strains, which is in sharp contrast to genetic variation in wild-derived mouse strains that is mostly unique [33]. Contrary to classical mouse inbred strains, where multiple subspecies contribute to the genetic make-up [13,34] and recent mouse strains, derived from different *Mus *species [35], laboratory rat strains are most likely descending from a single rat species, *Rattus norvegicus *[10].

An independent study using 42 microsatellites in German and Japanese wild-derived samples showed that the genetic profiles were quite divergent, partially owing to different geographic locations [36]. Our study involved only Dutch wild rats, suggesting that the inclusion of wild rats from different parts of the world could result in even more efficient SNP discovery, although it also remains to be demonstrated what proportion of the additional discovered alleles is present in the inbred strains and if a geographic bias for this exists.

When multiple SNPs are present per locus/amplicon, independent haplotypes can be discerned. The genetic variation identified here is mostly organized in a limited amount of haplotypes per locus (Table 4). Theoretically, an amplicon containing two or three SNPs can be represented by four and eight haplotypes, respectively, but in our dataset the vast majority of amplicons harboring multiple SNPs is represented by only two or three haplotypes (Table 4). Again, these observations suggest the existence of a common and small founding population with very limited haplotype diversity and/or a very narrow genetic bottleneck before inbred strain selection. The observed small genetic basis in a wide selection of laboratory rat strains does not mimic genetic variation in the human population and as a result, studies and pharmacological tests in rat models neglect potential modulatory effects caused by genetic variation. Although the use of F1 crosses and mosaic populations [37] could address this issue, our data suggests that wild-derived rats may be very useful to this end, since a large amount of all genetic variation present in a large selection of inbred strains, is already represented in a limited number of individuals. Therefore, it would be very interesting to investigate genetic variation in recently domesticated inbred [38] and outbred rats such as wild-type Groningen rats (WTG) [39]. Alternatively, careful selection of inbred strains based on genotyping data and subsequent random breeding may also expose the wild side of laboratory rats.

**Table 4:** Haplotype analysis in 36 strains for all SNP-containing amplicons

**number of haplotypes**												
	**2**	**3**	**4**	**5**	**6**	**7**	**8**	**9**	**10**	**11**	**12**	
**2 SNPs**	46	57	8									
**3 SNPs**	11	26	8	3	0	0	0					
**4 SNPs**	4	11	5	3	1	1	0	0	0	0	0	
**5 SNPs**	1	3	3	2	0	0	0	0	0	0	0	
**6 SNPs**	1	1	1	1	1	0	0	0	0	0	0	
**7 SNPs**	1	0	1	0	2	0	0	0	0	0	0	
**8 SNPs**	0	0	0	0	0	0	0	0	0	0	0	
**9 SNPs**	0	0	0	0	0	0	0	0	0	0	1	
**10 SNPs**	0	0	0	0	0	0	0	0	0	0	0	
**11 SNPs**	0	0	0	0	1	0	0	0	0	0	0	

**total**	64	97	27	9	5	1	0	0	0	0	1	204*

*) Total number of amplicons that contains at least two SNPs. Amplicons containing no SNPs or only indels were excluded from this analysis. Amplicons containing 1 SNP are also excluded, since two-state SNPs always give rise to two haplotypes.

## Conclusion

We describe a SNP discovery platform for the rat that is based on two steps. First, candidate SNPs are discovered by shotgun sequencing a wild rat, followed by genotyping laboratory strains of interest. We show that 84% of alleles in wild rats as compared to the sequenced Brown Norway rat genome are also represented in a set of 36 laboratory strains. Hence, the approach described here would be an efficient strategy for the discovery of novel informative SNPs in the laboratory rat. Inclusion of other wild samples, preferably from different locations in the world could result in an even more effective SNP discovery platform, as the three wild rats in our study, caught in relative close vicinity to each other, were already more polymorphic than the most polymorphic combination of carefully selected inbred strains. Based on the more than 34,000 genotyping datapoints obtained in this study, we postulate two things. First, laboratory rats originate from a single rat species, and inbred stains are relatively closely related with a limited number of haplotypes, reflecting known genetic bottlenecks in strain establishment. Second, wild rats have the potential to represent the degrees of genetic variation as present in the human population much more efficiently than a random selection of inbred strains. This makes them or wild-derived strains potentially well-suited for studying modulatory effects of genetic background variation on specific phenotypes, such as behavior or responses to drug treatment.

## Methods

### Genomic DNA isolation, shotgun library construction

Wild rat 1 (*Rattus norvegicus*) was caught in the canals of Utrecht and kindly provided by the Pest Control Service of the City of Utrecht (Utrecht, The Netherlands). Wild rat 2 was trapped in Gassel, a village located approximately 100 km south-east of Utrecht and was kindly provided by Tien Derks (Gassel, The Netherlands). Wild rat 3 was caught in a basement in Amsterdam, located 50 km north of Utrecht and kindly provided Romke Koch (Amsterdam, The Netherlands). Rat strains BN/Crl and Crl:Wistar (outbred) were obtained from Charles River The Netherlands. Liver samples of commonly used rat strains ACI/Ztm, BDE/Ztm, BDII/Ztm, BDIX/Ztm, BDV/Ztm, BH/Ztm, BN/Ztm, BS/Ztm, DA/Ztm, E3/Ztm, F344/Ztm, LE/Ztm, LEW/Ztm, LOU/CZtm, MNS/Ztm, MWF/Ztm, NAR/Ztm, OM/Ztm, PAR/Ztm, R33/Ztm, WC/Ztm, WF/Ztm, WKY/Ztm were provided by D.W. (Hannover Medical School, Germany) and liver samples of strains AO/OlaHsd, AUG/OlaHsd, BUF/SimRijHsd, COP/Hsd, DA/OlaHsd, LUDW/OlaHsd, PVG/OlaHsd, RP/AEurRijHsd, SHR/NHsd, SR/JrHsd, SS/JrHsd, WAG/RijHsd and 2 individuals of Hsd:SD (outbred) were kindly provided by Harlan (Horst, The Netherlands). Samples were lysed overnight in 20 ml lysis buffer, containing 100 mM Tris (pH 8.5), 200 mM of NaCl, 0.2% of SDS, 5 mM of EDTA, and 100 μg/ml of freshly added Proteinase K at 55°C under continuous rotation. Tissue debris was spinned down for 20 min at 10,000 × g and supernatant was transferred to a fresh tube. DNA was purified by phenol-chloroform extraction and precipitated by adding an equal volume of isopropanol, mixing and centrifugation for 20 min, 10,000 × g at 4°C. The supernatant was removed by gently inverting the tube and the pellets were washed with 70% ethanol and dissolved in 1000 μl water. The concentration was measured by Optical Densitometry at 260 nm.

### Wild rat-derived genomic library construction and shotgun sequencing

Sheared wild rat-derived genomic DNA of approximately 1–2 Kbp in size was cloned into the *Sma*I-site of pUC19. Fractions of the glycerol stock of the transformed library (*E. coli *DH10B) were plated on LB-plates containing 50 μg/ml ampicilin, 200 μg/ml IPTG, and 0.01% X-gal for standard blue/white screening on inserts. White colonies were picked in 20 μl water. Lysis occurred at 95°C for 10 min. 5 μl of 5× diluted lysate was used for the PCR reaction. For PCR, universal M13 primers were used, namely M13F: TGTAAAACGACGGCCAGT, M13R: AGGAAACAGCTATGACCAT. PCR, sequencing and cycling conditions were similar as for strain genotyping, described below. Sequencing was performed using universal M13 primers.

### PCR conditions for strain genotyping

PCR was carried out using a touchdown thermocycling program (92°C for 60 sec; 12 cycles of 92°C for 20 sec, 65°C for 20 sec with a decrement of 0.6°C per cycle, 72°C for 30 sec; followed by 20 cycles of 92°C for 20 sec, 58°C for 20 sec and 72°C for 30 sec; 72°C for 180 sec; GeneAmp9700, Applied Biosystems) and contained 30–50 ng genomic DNA, 0.2 μM of each forward primer and 0.2 μM of each reverse primer, 400 μM of each dNTP, 25 mM Tricine, 7.0% Glycerol (w/v), 1.6% DMSO (w/v), 2 mM MgCl_2_, 85 mM Ammonium acetate pH 8.7 and 0.2 U Taq Polymerase in a total volume of 10 μl.

### Sequencing reactions, purification, and analysis

PCR products were diluted with 25 μl water and 1 μl was directly used as template for the sequencing reactions. Sequencing reactions, containing 0.25 μl BigDYE (v3.1; Applied Biosystems, Foster City, CA, USA), 3.75 μl 2.5× dilution buffer (Applied Biosystems) and 0.4 μM universal M13 primer in a total volume of 10 μl, were performed using cycling conditions recommended by the manufacturer (40 cycles of 92°C for 10 sec, 50°C for 5 sec and 60°C for 120 sec). Of sequencing products, 5 μl was purified by ethanol precipitation in the presence of 40 mM sodium-acetate and analyzed on 96-capillary 3730XL DNA analyzers (Applied Biosystems), using the standard RapidSeq protocol. Sequences were analyzed for presence of heterozygous mutations using PolyPhred [40], followed by manual inspection of the polymorphic positions.

### Automation

All PCR and sequencing reactions were set up on a Tecan Genesis RSP200 liquid handling workstation, with a robotic and an 8-channel pipetting arm, an integrated 96-channel pipetting head (TEMO96, Tecan), and four integrated dual-384 well PCR blocks (Applied Biosystems).

### Mapping of shotgun reads and SNP discovery

Shotgun reads were assigned to positions in the RGSC 3.1 rat genome assembly using blat search [15]. Shotgun reads that complied with our mapping criteria, namely those having at least 80 identical bp for the best hit and no more than 60 identical bp for second blat hit were retained for further analysis. Blast nucleotide sequence alignments between shotgun read and corresponding genomic segment were used for discovery of single base variations (including single base indels). A site was treated as polymorphic only in the case when it has identical 5'- and 3'-flanks of at least 5 bp. A custom designed web-application was employed for manual chromatogram inspection and confirmation of a correct shotgun base-call for every polymorphic SNP locus. Primer design for resequencing was performed using a local web-interface [41] to the PRIMER3 program [42].

### Simulation model for wild rat-based SNP discovery

To estimate the number of SNPs to be discovered by the wild rat resequencing approach we performed computer simulations using the observed sample-specific polymorphism frequencies and the rat genome size of 2.48 Gbp as an input. We used a Monte-Carlo method for the placement of N 400-bp shotgun reads to the genome and calculated the total size of genome covered by N shotgun reads. To obtain a conservative estimate by assuming low heterozygosity in wild-derived strain the estimate of number of SNPs is given by product of covered genome size and polymorphism rate.

## Authors' contributions

BMGS contributed to the production of the results, supervised the ongoing of the study, and drafted the manuscript. VG contributed to the computational support of the results, and contributed to the writing of the manuscript. DZ contributed to the production of sequencing reads and initial analysis of the results. DW contributed to the preparation of samples for the study and revised the manuscript. HJH participated in the interpretation of the results and revision of the manuscript. EC outlined and supervised the study, and revised the manuscript. All authors read and approved the final manuscript.

## Supplementary Material

Additional File 1 Genotyping details; Detailed genotyping information, including allele information for the inbred and wild rat strains Click here for file
